# 905. Assessing Developmental and Emotional Needs in Pediatric Burn Patients Through Psychosocial Risk Evaluation

**DOI:** 10.1093/jbcr/irag033.409

**Published:** 2026-04-06

**Authors:** Alison J Burns, Steven A Kahn, Ashley B Hink, Betsy McMillan, Cecilia P Laverty, Tiffany Smith, Becca Anderson, Rohit Mittal

**Affiliations:** South Carolina Burn Center at Medical University of South Carolina, Charleston, SC; Medical University of South Carolina, Charleston, SC; Medical University of South Carolina, Charleston, SC; Medical University of South Carolina Shawn Jenkins Children's Hospital, Charleston, SC; South Carolina Burn Center, Charleston, SC; South Carolina Burn Center at Medical University of South Carolina, Charleston, SC; South Carolina Burn Center, Charleston, SC; South Carolina Burn Center at Medical University of South Carolina, Charleston, SC

## Abstract

**Introduction:**

Children with developmental delays are at high risk for burn injuries due to impaired safety awareness, vulnerability to non-accidental trauma and delayed communication. Once hospitalized, they face heightened psychosocial distress, including anxiety, and difficulty coping with invasive procedures. Despite these risks, psychosocial and developmental needs are often under-identified. The Psychosocial Risk Assessment in Pediatrics (PRAP) is a validated tool categorizing patients as low, moderate, or high risk by evaluating coping style, communication needs, and prior medical experiences. This project evaluated PRAP implementation in a pediatric burn unit to systematically identify developmental and emotional support needs and guide individualized care.

**Methods:**

This retrospective review of prospectively collected data was conducted January–August 2025 at an American Burn Association–verified burn center. All pediatric patients who sustained a burn injury under 18, admitted for more than 48 hours were screened with PRAP. Moderate-to-high risk scores (8–14 points) triggered adaptive care plans, psychosocial interventions, and therapy referrals. Follow-up screenings were performed weekly for inpatients or at the next clinic visit if discharged. Data included number screened, risk levels, developmental concerns, and outcomes at repeat screening.

**Results:**

PRAP screens were completed for 45 patients. Of these, 51% (n = 23) were moderate-to-high risk, prompting psychosocial interventions. Developmental delays were identified in seven children; three had pre-existing diagnoses, while four were newly identified during admission and referred for outpatient therapy - services they likely would not have received (Fig. 1). Mean PRAP scores decreased from 10 at initial screening (n = 45) to 8.5 at the second screen (n = 30) and 7.5 at the third (n = 18), reflecting a shift from moderate–high risk to low risk following interventions (Fig. 2).

**Conclusions:**

PRAP integration in burn care uncovered both known and previously unrecognized developmental delays. This allowed therapy referrals patients otherwise might have missed, providing support that can markedly improve developmental outcomes and justifying the added screening effort. Routine screening also guided adaptive care plans and showed clear clinical improvement, with mean risk scores declining across successive assessments. These findings support PRAP as an effective psychosocial risk-screening tool and highlight its role in advancing family-centered burn care across institutions.

**Applicability of Research to Practice:**

PRAP offers a structured approach to identifying psychosocial and developmental needs in pediatric burn patients. Integrating this screening into routine care facilitates early identification of unrecognized developmental delays and psychological distress, enabling timely, targeted interventions and improved coordination of outpatient care.

**Funding for the study:**

N/A.

